# Gut microbe-derived extracellular vesicles induce insulin resistance, thereby impairing glucose metabolism in skeletal muscle

**DOI:** 10.1038/srep15878

**Published:** 2015-10-29

**Authors:** Youngwoo Choi, Yonghoon Kwon, Dae-Kyum Kim, Jinseong Jeon, Su Chul Jang, Taejun Wang, Minjee Ban, Min-Hye Kim, Seong Gyu Jeon, Min-Sun Kim, Cheol Soo Choi, Young-Koo Jee, Yong Song Gho, Sung Ho Ryu, Yoon-Keun Kim

**Affiliations:** 1 Department of Life Sciences, Pohang University of Science and Technology (POSTECH), Pohang, Republic of Korea; 2 Department of Interdisciplinary Biosciences and Biotechnology, POSTECH, Pohang, Republic of Korea; 3 Department of Medicine, Ewha Womans University School of Medicine and Ewha Institute of Convergence Medicine, Seoul, Republic of Korea; 4 Department of Internal Medicine, Ulsan University College of Medicine, Seoul, Republic of Korea; 5 Korea Mouse Metabolic Phenotyping Center, Lee Gil Ya Cancer and Diabetes Institute, and Division of Endocrinology, Gil Medical Center, Gachon University, Incheon, Republic of Korea; 6 Department of Internal Medicine, Dankook University College of Medicine, Cheonan, Republic of Korea

## Abstract

Gut microbes might influence host metabolic homeostasis and contribute to the pathogenesis of type 2 diabetes (T2D), which is characterized by insulin resistance. Bacteria-derived extracellular vesicles (EVs) have been suggested to be important in the pathogenesis of diseases once believed to be non-infectious. Here, we hypothesize that gut microbe-derived EVs are important in the pathogenesis of T2D. *In vivo* administration of stool EVs from high fat diet (HFD)-fed mice induced insulin resistance and glucose intolerance compared to regular diet (RD)-fed mice. Metagenomic profiling of stool EVs by 16S ribosomal DNA sequencing revealed an increased amount of EVs derived from *Pseudomonas panacis* (phylum Proteobacteria) in HFD mice compared to RD mice. Interestingly, *P. panacis* EVs blocked the insulin signaling pathway in both skeletal muscle and adipose tissue. Moreover, isolated *P. panacis* EVs induced typical diabetic phenotypes, such as glucose intolerance after glucose administration or systemic insulin injection. Thus, gut microbe-derived EVs might be key players in the development of insulin resistance and impairment of glucose metabolism promoted by HFD.

Coelomate animals possess an internal body cavity surrounding the gut and other organs and have coevolved with a diverse range of symbiotic gut microbes collectively known as the gut microbiota^1^. The gut microbiota is involved in the regulation of systemic immune responses, and influence multiple host metabolism^2^,^3^,^4^. The increased incidence of gut dysbiosis (an imbalance in the gut microbes leading to disease) in westernized countries over several decades is associated with metabolic diseases such as obesity and type 2 diabetes (T2D)^5^,^6^,^7^,^8^. T2D, one of the most well-known carbohydrate metabolism disorders, accounts for almost 90% of adult diabetes cases and is characterized by insulin resistance and low-grade inflammation^9^,^10^,^11^,^12^. Although commensal gut microbes have been implicated in the pathogenesis of T2D promoted by a high fat diet (HFD), the mechanism underlying this phenomenon is still unclear.

Host and gut microbes coproduce a large array of materials during the metabolism of food, many of which play critical roles in shuttling information between host cells and the microbes. Extracellular vesicles (EVs) were found about 30 years ago when EVs in multivesicular bodies were observed in reticulocytes and released into the extracellular space^13^,^14^. It is now known that both bacterial and eukaryotic cells release EVs as a means of intercellular communication, thereby influencing neighboring and distant cells^15^,^16^,^17^. Bacteria-derived EVs, also called nanovesicles, are spherical bi-layered phospholipids with diameters ranging from 20 to 100 nm. Nanovesicles are produced ubiquitously by all gram-negative bacteria and also by some gram-positive bacteria^18^,^19^. Previous biochemical studies revealed that gram-negative bacteria-derived EVs are composed of outer membrane proteins, lipopolysaccharide (LPS), outer membrane lipids, periplasmic proteins, DNA, RNA, and other factors associated with virulence^18^. The role of infectious agents in the etiology of diseases once believed to be non-infectious is increasingly being recognized^20^. As a candidate for the causative agents of these diseases, our previous studies have shown that EVs from indoor dust and from gram-positive bacteria induce neutrophilic pulmonary inflammation^21^,^22^.

In this study, we hypothesized that EVs-mediated cross-talk between the host and gut microbiota may underlie the development of T2D in a fat-rich dietary regimen. Here, we showed that *in vitro* administration of microbe-derived EVs induced insulin resistance including impairment of insulin signaling and decrease in GLUT4 translocation in myotubes. Moreover, these EVs also induced diabetic phenotypes in mice, such as glucose intolerance after glucose administration and insulin injection.

## Methods

### Mice

C57BL/6J (6–8-wk-old) mice from the Jackson Laboratory were used. All animal experiments were approved by POTECH Animal Use and Care Committee (Permit Number: 2012-01-0020) and performed in accordance with the Guide for the Care and Use of Laboratory Animals published by Animal and Plant Quarantine Agency, Ministry of Agriculture, Food and Rural Affairs, Korea.

### Isolation of stool EVs

We placed each group of mice on an RD, or an HFD containing 60% fat for 12 weeks and collected stool samples at 12 weeks. Stool samples were dissolved in Phosphate Buffered Saline (PBS) and centrifuged at 5, 20, and 340 *g* for 5 min each. Supernatant fractions were pelleted once at 10,000 *g* for 30 min and then filtered through a 0.45-μm syringe filter (Sartorius Stedim Biotech, Goettingen, Germany) followed filtration through a 0.22-μm syringe filter (Sartorius Stedim Biotech, Goettingen, Germany). The filtrates were then subjected to density-gradient centrifugation in a Beckman ultracentrifuge (Beckman Coulter, Fullerton, USA) at 100,000 *g* for 2 h at 4 °C. Fraction between 10% and 40% OptiPrep solution (Sigma, St. Louis, USA) was taken, and EVs were prepared by centrifugation at 150,000 *g* for 2 h at 4 °C using a Beckman ultracentrifuge. EVs were diluted in PBS and stored at –80 °C. The protein concentration of EVs was assessed by a BCA assay (Thermo Fisher Scientific, Waltham, USA).

### Preparation of bacterial EVs

Bacterial EVs were prepared as described previously^18^. *P. cedrina* was grown at 30 °C and *P. panacis* at 25 °C in Luria-Bertani broth. Culture media were pelleted twice at 10,000 *g* for 30 min. Supernatant fractions were filtered through a 0.45-μm bottle-top filter (Sigma, St. Louis, USA), and the eluted samples were enriched using QuixStand™ (GE Healthcare, Little Chalfont, UK). Concentrated samples were subsequently filtered through a 0.22-μm bottle-top filter (Sigma, St. Louis, USA). The filtrates were then subjected to centrifugation in a Beckman ultracentrifuge at 150,000 *g* for 2 h at 4 °C. The protein concentration of EVs was assessed by a BCA assay (Thermo Fisher Scientific, Waltham, USA).

### Transmission electron microscopy

EVs (50 μg/mL of total protein) were placed on 300-mesh copper grids (Electron Microscopy Sciences, Hatfield, USA) and stained with 2% uranyl acetate for 12 h. Images were obtained by using a JEM1011 microscope (JEOL, Akishima, Japan) at an accelerating voltage of 100 kV.

### Dynamic light scattering

Diameter of EVs (5 μg/mL of total protein) was measured by using a Zetasizer Nano S (Malvern Instruments, Malvern, UK) equipped with a 633-nm laser line at a scattered intensity of 10 × 30 s.

### Detection of lipopolysaccharide (LPS) and lipoteichoic acid (LTA)

LPS and LTA were detected by using primary antibodies against lipid A (Abcam, Eugene, USA) and lipoteichoic acid (Abcam, Eugene, USA).

### DNA extraction, bacterial 16S rDNA gene amplification and pyrosequencing analysis

DNA was extracted from bacteria and EVs in stools using a stool DNA extraction kit (Bioneer, Daejeon, Korea). Bacterial genomic DNA was amplified with 27F (5′-AGAGTTTGATCCTGGCTCAG-3′) and 518R (5′-ATTACCGCGGCTGCTGG-3′) primers, which are specific for V1-V3 hypervariable regions of 16S rDNA gene. The libraries were prepared using PCR products according to the GS FLX titanium library prep guide and quantified using a Picogreen assay (Invitrogen, Waltham, USA). After PCR products extracted and quantified, equimolar ratios from each mixture were pooled and sequenced on a 454 Life Sciences Genome Sequencer FLX system (Roche, Basel, Switzerland) according to the manufacturer’s recommendations.

### Taxonomic assignment

Raw pyrosequencing reads obtained from the sequencer were filtered according to the barcode and primer sequences using sffinfo script (Roche, Basel, Switzerland). Preliminary quality control steps included the removal of sequences shorter than 300 nt. To select 16S rDNAs, all the sequence reads were compared to the EzTaxon database^23^,^24^ by BLASTN^25^. Sequence reads that had a similar sequence with more than 100 bit score and less than 1.0 E-value were admitted as partial 16S rDNA sequences. Taxonomy-based analyses were performed using EzTaxon. From the database, five of the most similar sequences for each sequence read were found by their bit scores by using the BLASTN program. Among these five sequences, similarity with the sequence read was calculated by ClustalW^26^, and the taxonomy of sequences with the highest similarity was assigned to the sequence read.

### L6 myotubes culture

L6 myotubes were differentiated and cultured as described previously^27^. L6 myotubes were grown in Alpha Minimum Essential Medium (α-MEM) containing 10% fetal bovine serum (FBS) and 1% penicillinstreptomycin at 37 °C, 5% CO_2_ in a humidified incubator. Differentiation of L6 myotubes was induced by incubation in 2% FBS containing α-MEM for 1 week.

### 3T3-L1 adipocytes culture

The 3T3-L1 pre-adipocyte cell line was maintained in Dulbecco’s Modified Eagle’s Medium (DMEM) containing 10% fetal bovine serum (FBS) and 1% penicillinstreptomycin. The procedure of adipocyte differentiation was performed as described previously^28^.

### Analysis of the insulin signaling pathway

To prepare total cell lysates, plated cells were lysed with lysis buffer containing 40 mM HEPES, 120 mM NaCl, 1 mM EDTA, 10 mM pyrophosphate, 10 mM glycerophosphate, 50 mM NaF, 1.5 mM Na_3_VO_4_, 1 mM PMSF, 5 mM MgCl_2_, 0.5% Triton X-100, and protease inhibitor mixture. Following SDS-PAGE and transfer to a nitrocellulose membrane, each molecular size of nitrocellulose membrane was incubated with primary antibodies (1:1000) overnight at 4 °C. For evaluation of the insulin signaling pathway, the following antibodies were used: anti-tIRS1 (EMD Millipore, Billerica, USA), anti-pIRS1 (Cell Signaling Technology, Danvers, USA), anti-tAKT (Santa Cruz Biotechnology, Dallas, USA), anti-pAKT (Cell Signaling Technology, Danvers, USA), and anti-actin (MP Biochemicals, Santa Ana, USA).

### Glucose uptake measurement

2-Deoxyglucose uptake was determined as described previously^29^. L6 myotubes were serum starved for 2 h before treatment with EVs (10 μg of total protein) for 4 h. The medium was replaced with 1 mL of Kreb’s buffer and cells were stimulated with 5 nM insulin for 10 min at 37 °C. Glucose uptake was measured by adding 0.1 mCi/mL of 2-deoxy[^14^C]glucose (final concentration: 0.1 mM). After 10 min, assays were terminated by washing with cold PBS twice. Cells were lysed in 0.5 mL of lysis buffer containing 0.5 N NaOH and 0.1% SDS, and cell lysates were subjected to liquid scintillation counting.

### o-Phenylenediamine dihydrochloride (OPD) assay

A colorimetric assay of surface GLUT4myc was performed using the anti-Myc antibody (1:100) as described previously^30^. EVs (10 μg of total protein) were administrated to L6 myotubes for 4 h following 2 h of serum starvation. The cells were washed once with PBS and then incubated with anti-Myc antibody (EMD Millipore, Billerica, USA). After incubation with the primary antibody, peroxidase-conjugated rabbit anti-mouse IgG (1:1000) was added for 30 min at 4 °C. The cells were washed and 1 mL of OPD reagent was added. The colorimetric reaction was stopped by adding 0.25 mL of 3N HCl. After 10 min, the optical absorbance of the supernatant was measured at 492 nm.

### *In vivo* metabolic studies

EVs (1 μg of total protein) were orally administered once every 2 days for 1 month. For the glucose tolerance test (GTT), mice were initially fasted for 12 h. Then, 1 gram of dextrose (Sigma, St. Louis, USA) per kilogram body weight was administered intraperitoneally, and blood glucose concentrations were subsequently measured at the indicated time points. For the insulin tolerance test (ITT), the mice were fasted for 6 h, followed by intraperitoneal injection of 0.5U insulin (Sigma, St. Louis, USA) per kilogram body weight. Blood glucose concentrations were measured at indicated time points using a glucometer (Abbott Diabetes Care, Alameda, USA.). The blood used for both GTT and ITT was withdrawn from the tail vein of mice.

### *In vivo* fluorescent imaging

Bacteria or bacterial EVs were incubated with 5 μM Cy7 mono NHS ester (GE Healthcare, Little Chalfont, UK) for 1 h at 37 °C. Cy7 mono NHS ester-labbeled samples were isolated using ultracentrifugation. Then, bacteria or bacterial EVs (20 μg of total protein) were administered by gavage to each mouse that had been fasted overnight. At the indicated time point, whole body images were obtained at 780–800 nm wavelength using IVIS® spectrum CT (SelectScience, Wilmslow, UK). After 12 h, the mice were sacrificed and Cy7 fluorescence was quantified in tissues including the liver, fat, and muscle.

### Two-photon microscopy

*P. panacis*-derived EVs were incubated with 5–10 μM DiO (Invitrogen, Waltham, USA) for 30 min at 37 °C. The mice were fasted overnight and the large intestines were pulled out. Both sides of the large intestine were sealed, and then fluorescent-labeled EVs (10 μg of total protein) were administered into the intestinal lumen. Images were taken 10 min after the application, by using a TCS SP5 microscope (Leica Microsystems, Wetzlar, Germany).

### Immunohistochemistry

Extracted liver, adipose tissue, skeletal muscle, and large intestine were embedded in an OCT compound (Sakura Finetek, Radnor, USA), frozen, and cut with a CM-3050S (Leica Microsystems, Wetzlar, Germany) into 0.2-μm-thick sections. For the detection of EVs, blocking was performed using 0.05% horse serum. Primary antibodies (1:100) were diluted in blocking buffer and added to samples overnight at 4 °C. After three washes, GFP-conjugated secondary antibodies (1:5000) were added, and the sections were incubated for 1 h at 37 °C. Subsequently, 4′,6-diamidino-2-phenylindole (DAPI) staining was performed. Samples were then imaged using a TCS SP5 microscope (Leica Microsystems, Wetzlar, Germany).

### Statistics

All statistical analyses consisted of unpaired, two-tailed Student’s t-tests using Excel (Microsoft, Redmond, USA), followed by Bonferroni’s post hoc test using GraphPad Prism5 (GraphPad Software, San Diego, USA). *P* < 0.05 was considered to be significant. All data are expressed as means ± SD.

## Results

### Characterization of stool EVs from HFD- and RD-fed mice

**Figure 1:**
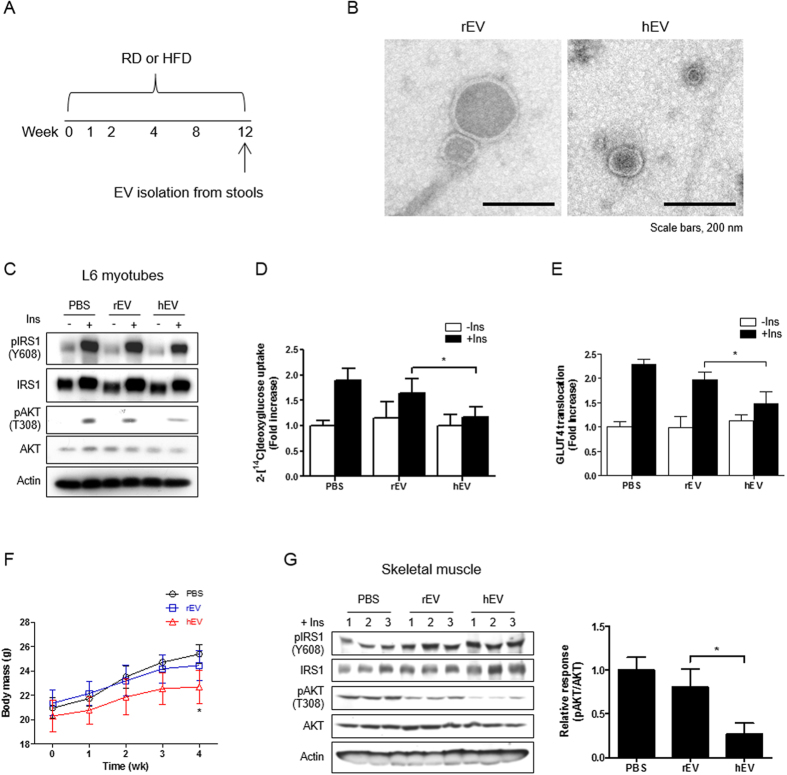
Insulin resistance and glucose intolerance are induced by stool EVs isolated from HFD-fed mice. (**A**) An experimental protocol for isolating stool EVs in HFD- and RD-fed mice. (**B**) Transmission electron microscopic (TEM) images of stool EVs (hEV vs. rEV). (**C**) Western blot data of insulin signaling molecules in L6 myotubes after treating stool EVs (hEV or rEV), with or without insulin (Ins). (**D**) 2-Deoxy[^14^C]glucose uptake in L6 myotubes in response to stool EVs (hEV or rEV), with or without Ins treatment. *P < 0.05. (**E**) GLUT4 translocation to the membrane of L6 myotubes in response to stool EVs (hEV or rEV), with or without Ins. *P < 0.05. (**F**) Change of mouse body weight at the indicated day after the oral administration of stool EVs (hEV or rEV) (n = 8 mice per group). (**G**) Phosphorylation of IRS1 and AKT in skeletal muscle from hEV- or rEV-administered mice. *P < 0.05.

To assess changes in gut microbe-derived EVs in response to a change in diet, mice were fed with HFD or RD for 12 weeks (Fig. 1A). EVs were isolated from stools collected from the 2 groups, HFD or RD. The size of EVs from HFD-fed mice stools (hEV) showed drastic decrease from that of EVs from RD-fed mice stools (rEV) (Fig. 1B and S1A). Protein profiling evaluation showed that the protein pattern in hEV was different from rEV (Figure S1B). In addition, hEV had more LPS, but less LTA vs. rEV (Figure S1C). These findings suggest that a change in diet from RD to HFD regimen resulted in a change in the characteristics of gut microbial EVs.

### *In vitro* effects of stool EVs enhanced by HFD on the insulin signaling and glucose metabolism

To evaluate the *in vitro* effects of gut microbial EVs on the insulin resistance, skeletal muscle cells (myotubes) were treated with hEV and rEV, with and without insulin. The expression of phosphorylated AKT (pAKT), which enhanced by insulin treatment, was inhibited in myotubes by the treatment of hEV vs. rEV (Fig. 1C). Furthermore, insulin-stimulated glucose uptake by these cells was also decreased by hEV treatment as compared to rEV treatment (Fig. 1D). In addition, an *in vitro* assay after insulin stimulation displayed the blunted increase in GLUT4 translocation to the membrane of myotubes treated with hEV vs. rEV (Fig. 1E). Taken together, these findings suggest that EVs enhanced by HFD interfere with insulin signaling and impair glucose uptake in skeletal muscle.

### *In vivo* effects of stool EVs enhanced by HFD on the insulin signaling and glucose metabolism

To address the *in vivo* effects of EVs from HFD-fed mice stools on the glucose metabolism, EVs, isolated 12 weeks after the initiation of HFD, were administered orally, and then insulin and glucose tolerance tests were performed. This study showed that body weight gain was blunted in mice administered with hEV compared to rEV (Fig. 1F). In addition, the decline of blood glucose after glucose administration and after insulin injection was blunted in hEV-administered mice vs. rEV-administered mice (Figure S2). Furthermore, the expression of pAKT in skeletal muscle was decreased in hEV-treated mice compared to rEV-treated mice (Fig. 1G). Collectively, these findings indicate that EVs enhanced by HFD can induce insulin resistance and glucose intolerance in murine skeletal muscle.

### Compositional changes of bacteria and bacteria-derived EVs in stools according to diet change

**Figure 2:**
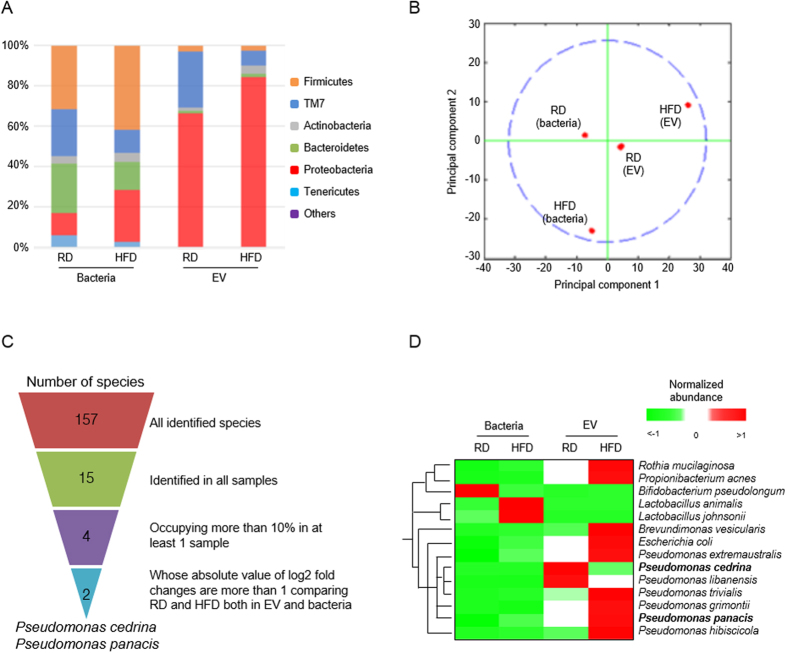
Stool metagenomic analysis indicates that HFD increases the composition of EVs derived from phylum Proteobacteria, including *Pseudomonas panacis*. For all figures, stools were isolated from mice after 12 weeks of HFD or RD. (**A**) Relative abundance (% of total 16S rDNA gene sequences) of gut microbes and gut microbe-derived EVs at the phylum level. (**B**) Principal component analysis (PCA) of bacteria and stool EVs. Note the change of EVs composition is greater than that of bacterial composition, since EVs composition is separated with principal component 1. (**C**) Four filters were used to select the most essential species, in which EVs were significantly changed after HFD, and then *P. cedrina* and *P. panacis* were selected. (**D**) Heatmap plot of stool bacteria and bacterial EVs at the species level. Note that the only species occupying more than 1% in at least one sample were included.

The composition of gut microbes and gut microbe-derived EVs in stools were evaluated using 16S rDNA sequencing in mice fed with HFD or RD, as a control, 12 weeks after the initiation of HFD. The composition of bacteria and bacterial EVs was profiled at the levels of phylum, class, order, family, genus, and species (Tables S1–S3 and Figure S3). In agreement with a previous report^31^, HFD induced a change in bacterial composition at the phylum level, primarily an increase in Firmicutes and Proteobacteria, and a decrease in Bacteroidetes. Notably, the composition of bacterial EVs differed from that of bacteria (Fig. 2A). Diet change from RD to HFD induced more drastic changes in the composition of gut-microbial EVs vs. gut microbes (Fig. 2B). To select candidate EVs, as a cause of T2D promoted by HFD, data from 16S rDNA sequencing were filtered by four sequential steps, as shown in Fig. 2C. This study showed that the composition of *P. panacis*-derived EVs dramatically increased by HFD and were selected as a candidate causative agent, whereas that of *P. cedrina*-derived EVs decreased and selected as a control (Fig. 2D).

### Characterization of EVs derived from *P. cedrina* and *P. panacis*

**Figure 3:**
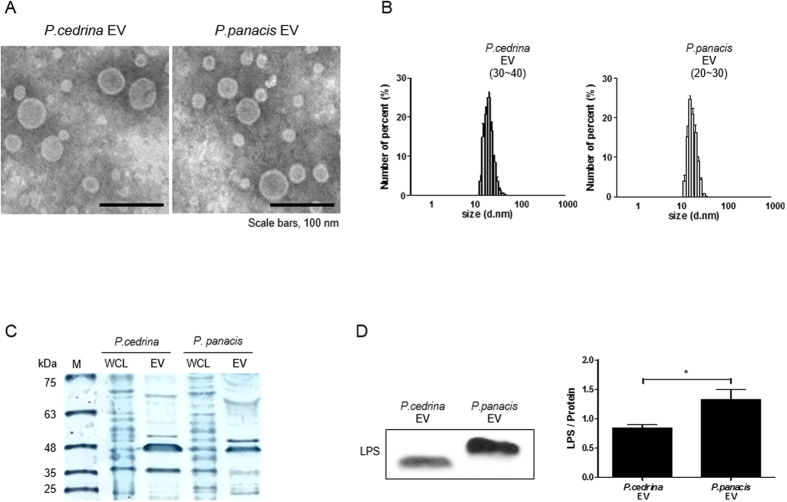
Characterization of EVs derived from *P. cedrina* and *P. panacis.* (**A**) Transmission electron microscopy (TEM) image of *P. cedrina* EVs and *P. panacis* EVs. (**B**) Size of *P. cedrina* EVs and *P. panacis* EVs. (**C**) SDS-PAGE analysis of *P. cedrina* whole cell lysate (WCL)*, P. cedrina* EVs, *P. panacis* whole cell lysate (WCL), and *P. panacis* EVs. (**D**) Western blot data (left panel) and ELISA assay (right panel) of lipid A in *P. cedrina* EVs and *P. panacis* EVs.

To characterize EVs from *P. cedrina* and *P. panacis*, we cultured these two bacteria, and isolated EVs from the culture supernatants of culture media. The present study showed that *P. cedrina*- and *P. panacis*-derived EVs were spherical in shape (Fig. 3A), and the size of these two EVs were 30–40 nm and 20–30 nm in diameter, respectively (Fig. 3B). In addition, this study showed that protein profile is different between whole cell lysate (WCL) and EVs, and that protein profile was also different between EVs from the two bacteria (Fig. 3C). Moreover, the amount of LPS was higher in *P. panacis* EVs than *P. cedrina* EVs (Fig. 3D). These data suggest that there is a compositional difference between *P. cedrina* and *P. panacis* EVs.

### *In vitro* effects of *P. cedrina*- and *P. panacis*-derived EVs on the insulin signaling and glucose uptake in skeletal muscle cells

**Figure 4:**
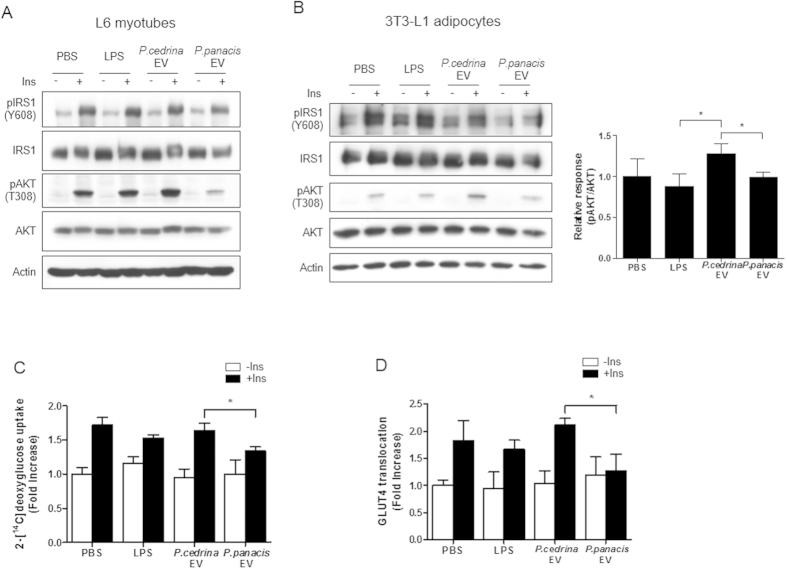
*P. panacis* EVs interfere with insulin signaling in both myotubes and adipocytes, and impair glucose uptake in myotubes. (**A**) Immunoblot analysis of the insulin signaling molecules in L6 myotubes after the treatment of LPS, *P. cedrina* EVs, or *P. panacis* EVs, with or without insulin (Ins). (**B**) Werstern blot data of the insulin signaling molecules in 3T3-L1 adipocytes after the application of LPS, *P. cedrina* EVs, or *P. panacis* EVs, with or without Ins. *P < 0.05 vs. *P. cedrina* EVs. (**C**) 2-Deoxy[^14^C]glucose uptake in L6 myotubes in response to LPS, *P. cedrina* EVs, or *P. panacis* EVs, with or without Ins. *P < 0.05. (**D**) GLUT4 translocation to the membrane of L6 myotubes after the treatment of LPS, *P. cedrina* EVs, or *P. panacis* EVs, with or without Ins. *P < 0.05.

To evaluate the *in vitro* effect of *P. cedrina*- and *P. panacis*-derived EVs on the insulin signaling and glucose uptake in skeletal muscle, LPS, *P. cedrina* EVs, and *P. panacis* EVs were treated to myotubes. This study showed that the insulin-induced expression of pAKT, a signaling molecule of insulin, was abolished by treatment with *P. panacis* EVs, but unaffected by *P. cedrina* EVs, LPS, or PBS (Fig. 4A). Additionally, the insulin-induced expression of pAKT in adipocytes was decreased by treatment with *P. panacis* EVs or LPS, compared to *P. cedrina* EVs (Fig. 4B). In contrast, the EGF-induced expression of signaling molecules in myotubes was found to be unaffected by the treatment of *P. panacis* EVs (Fig. S4). These findings suggest that the inhibitory effect of *P. panacis* EVs on transduction pathways is specific to the insulin signaling pathway in insulin-responsive cells. The effect of *P. panacis* EVs on the glucose uptake in skeletal muscle was assessed by using an *in vitro* glucose uptake assay. This study revealed that an insulin-stimulated glucose uptake was blunted in myotubes by the treatment with *P.* panacis EVs, but not with *P. cedrina* EVs or LPS (Fig. 4C). Moreover, the insulin-enhanced GLUT4 translocation in myotubes was inhibited by the treatment of *P. panacis* EVs, but not of *P. cedrina* EVs or LPS (Fig. 4D). Taken together, these data indicate that *P. panacis* EVs can interfere with insulin signaling in both skeletal muscle and adipose tissue, and also glucose uptake in skeletal muscle via the inhibition of GLUT4 translocation.

### *In vivo* effects of *P. cedrina*- and *P. panacis*-derived EVs on the insulin signaling and glucose metabolism

**Figure 5:**
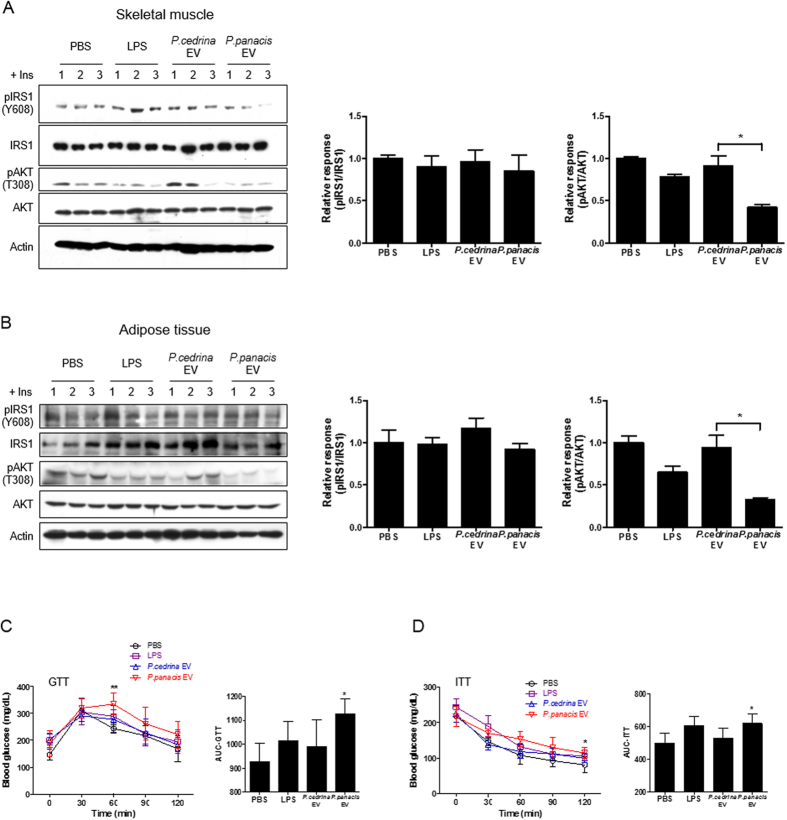
*P. panacis* EVs induce insulin resistance and diabetic phenotypes in RD-fed mice. For all figures, LPS, *P. cedrina* EVs, and *P, panacis* EVs were administered orally to mice once every 2 days for 4 weeks. All mice were fed with RD. (**A**) Phosphorylation of IRS1 and AKT in skeletal muscle after the last injection. *P < 0.05. (**B**) Immunoblot analysis of insulin signaling molecules in the adipose tissue after the last application. *P < 0.05. (**C**) Glucose tolerance test (GTT). GTT was performed in mice 12 h fasting after the last application (n = 4 mice per group). *P < 0.05 vs. the other groups. (**D**) Insulin tolerance test (ITT). ITT was performed in mice 6 h fasting after the last application (n = 5 mice per group). *P < 0.05 vs. the other groups.

To address the *in vivo* effects of *P. cedrina*- and *P. panacis*-derived EVs on the glucose metabolism and insulin sensitivity, LPS, *P. cedrina* EVs, and *P. panacis* EVs were administered to the mouse stomach. Body weight significantly decreased in mice that received *P. panacis* EVs or LPS compared to *P. cedrina* EVs (Figure S5). Furthermore, the insulin signaling pathway was blocked at the pAKT level in both skeletal muscle and adipose tissue, isolated from *P. panacis* EVs-received mice, when compared to *P. cedrina* EVs-received mice (Fig. 5A,B). To evaluate whether insulin resistance was induced in response to *P. panacis* EVs, we performed insulin-tolerance test (ITT) and glucose-tolerance test (GTT). Blood glucose levels exhibited a blunt response to both glucose loading and insulin injection in mice administered with *P. panacis* EVs vs. *P. cedrina* EVs and LPS (Fig. 5C,D). Taken together, these data imply that *P. panacis* EVs can impair glucose metabolism by inducing insulin resistance in both skeletal muscle and adipose tissue.

### Absorption and distribution of *P. panacis* bacteria and *P. panacis*-derived EVs after oral administration

**Figure 6:**
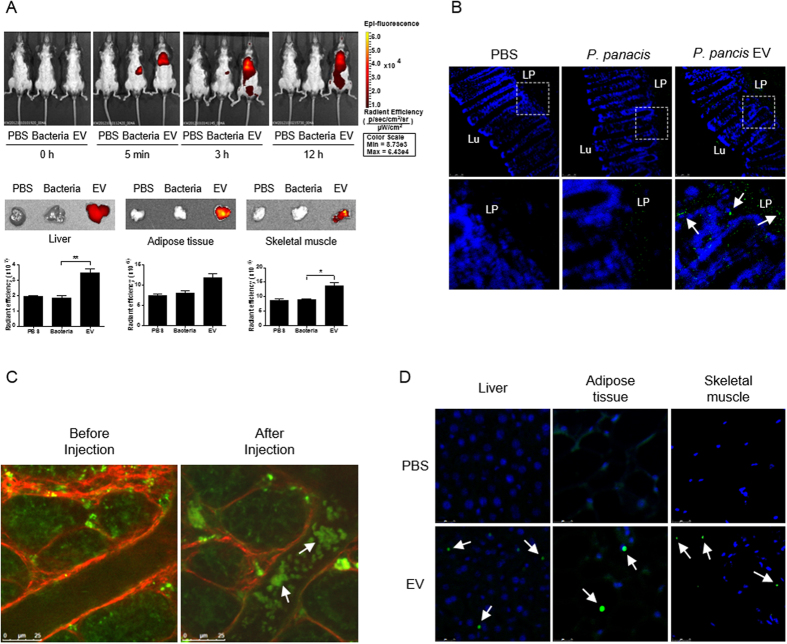
Absorption and distribution of *P. panacis* bacteria and *P. panacis* EVs after oral administration. (**A**) *In vivo* fluorescent whole body image of *P. panacis* bacteria and *P. panacis* EVs trafficking in C57BL/6J mice before and after the administration by gavage after overnight fasting (upper panel). Lower panel indicates the images of insulin-sensitive organs (liver, adipose tissue, and skeletal muscle), which were extracted from mice 12 h after the oral administration. *P < 0.05, **P < 0.01. (**B**) Immunohistochemistry using *P. panacis* EVs-reactive polyclonal antibodies. Images were taken from the large intestine 10 minutes after the surgical injection of *P. panacis* bacteria and *P. panacis* EVs. *P. panacis* EVs-reactive antibodies (green dots indicated by white arrows) are seen in the lamina propria (LP) of the large intestine. Lu: intestinal lumen. (**C**) *In vivo* two-photon image of the large intestine before and 10 minutes after the luminal administration of *P. panacis* EVs. EVs (green dots indicated by white arrows) are observed inside the blood vessels in the intestinal lamina propria. Scale bar = 25 μm. (**D**) Immunohistochemistry using *P. panacis* EVs-reactive polyclonal antibodies. *P. panacis* EVs-reactive antibodies (green dots indicated by white arrows) are observed in liver, adipose tissue, and skeletal muscle, extracted from mice 12 h after the oral administration of *P. panacis* EVs. Scale bar = 25 μm.

An *in vivo* imaging study was performed to evaluate whether gut microbes and their secreting EVs were absorbed past the intestinal barriers and moved to target organs, including the liver, adipose tissue, and skeletal muscle, after the oral administration of *P. panacis* bacteria and *P. panacis*-derived EVs. *In vivo* whole body imaging showed that the bacteria itself did not infiltrate through the gut to other organs; however, in mice administered with bacterial EVs, EVs were present in the heart and lung areas 5 minutes after the application (Fig. 6A, upper panel). In addition, imaging data 12 h after the application showed the systemic distribution of EVs, including into the insulin-responsive organs, such as the liver, adipose tissue, and skeletal muscle (Fig. 6A). It was further investigated whether *P. panacis* EVs infiltrated the gut barrier and then invaded the target organs. First, we marked a section of the large intestine, secured the ends of the sections, and then administered *P. panacis* bacteria and *P. panacis* EVs. Immunohistochemistry showed that EVs were present in the lamina propria, whereas the bacteria were not (Fig. 6B). In addition, two-photon microscopy showed that EVs were present in the capillaries of the lamina propria after this application (Fig. 6C). Moreover, immunohistochemical staining using *P. panacis* EVs-reactive polyclonal antibodies revealed that *P. panacis* EVs, after the oral administration of EVs, were present in the liver, adipose tissue and skeletal muscle (Fig. 6D). To sum up, these data indicate that bacteria-derived EVs, but no bacteria, infiltrate through the intestinal barrier and then distribute into the insulin-responsive tissues.

## Discussion

Gut microbes are increasingly being accepted as an environmental factor that affects host metabolism and contributes to metabolism-associated pathologic conditions, such as obesity, T2D, and cardiovascular disease. However, the mechanism(s) by which gut microbes cause T2D is still unclear. The data presented here showed that the HFD enhanced the release of EVs in the gut from microbes (especially phylum Proteobacteria) containing LPS. Furthermore, the composition of EVs from *P. panacis*, belonging to Proteobacteria, was increased by the HFD, and *P. panacis* EVs caused insulin resistance and glucose intolerance in mice.

Recent from the literature highlighted a tight and coordinated connection between gut microbes and host metabolism, energy utilization, and storage^31^. The gut microbial community comprises trillions of microorganisms. Cani *et al.*^32^ reported that a 4-week HFD increases the composition of LPS-expressing bacteria in the gut and leads to a two- to three-fold elevation in plasma LPS, a so-called metabolic endotoxemia. As a consequence, chronic inflammation induced by low-grade endotoxemia induces insulin resistance and glucose intolerance. In addition, the same group provided evidence that antibiotic treatment changes the composition of gut microbiota and reduces fecal and plasma LPS levels^33^. Consistent with these results, our current metagenomic data showed that the composition of EVs-derived from LPS-expressing Proteobacteria increased in HFD-fed mice versus RD-fed mice. Moreover, the present study revealed that stool EVs from HFD-fed mice induced insulin resistance and glucose intolerance in both *in vitro* and *in vivo* models. Taking the presence of LPS in EVs into consideration, these data suggest that LPS-expressing EVs derived from gut microbes are key mediators of the development of insulin resistance and glucose intolerance promoted by HFDs.

A culture-independent metagenomic approach has provided new insight into the complex interactions between mammalian hosts and gut microbes^34^. Genetically obese *ob/ob* mice are hyperphagic as a result of a mutation in the gene that encodes the satiety-promoting hormone leptin^35^. The gut microbiota of these mice contains more Firmicutes and fewer Bacteroidetes than their lean wild-type littermates^35^. Similar changes have also been observed in the gut microbiota of obese humans^7^ and in people whose body weight decreased after a Roux-en-Y gastric bypass procedure^36^. The present study also showed that the composition of Firmicutes increased, whereas that of Bacteroidetes decreased, in the gut of HFD-fed mice versus RD-fed mice. In summary, these findings indicate that the composition of gut microbes is dependent on both genetic and environmental factors.

Human data indicate that gut microbes are involved in the pathogenesis of T2D^8^. In the present study, we propose that gut microbe-derived EVs play a key role in intracellular communication between gut microbes and hosts in glucose metabolism. This study showed that the composition of gut microbe-derived EVs significantly differed from that of gut microbes, and that diet change from RD to HFD induced more drastic changes in the composition of bacterial EVs versus bacteria. Among gut microbe-derived EVs, we found that the composition of *P. panacis* EVs markedly increased in HFD-fed mice versus RD-fed mice. The present data showed that among gut microbe-derived EVs, the *P. panacis* EVs composition markedly increased in HFD-fed mice versus RD-fed mice. Interestingly, this study showed that *P. panacis* EVs induced insulin resistance in both skeletal muscle and adipose tissue, thereby promoting glucose intolerance in skeletal muscle. Taken together, these data suggest that gut microbe-derived EVs promoted by an HFD can induce T2D via the induction of insulin resistance in insulin-responsive organs.

In the present study, we propose that gut microbe-derived EVs are key communication messengers between gut microbes and hosts in the regulation of glucose metabolism. It is increasingly evident that gut microbes may shape host metabolic and immune network activities and ultimately influence the development of obesity and diabetes. Although much solid evidence indicates that excess energy intake, such as HFDs, can affect microbial composition, the mechanism by which gut microbes contribute to insulin resistance and thereby T2D is still unclear. Two hypotheses to explain the mechanisms of insulin resistance and T2D are as follows: (1) LPS is taken up with dietary fats in chylomicrons^37^ or (2) LPS reaches the circulation because the gut is more permeable in susceptible mice^32^. Plasma LPS levels rise with increase in fat intake in mice^32^ and humans^38^. Animal studies showed that targeted deletion of fatty acid synthase in the mouse gut epithelium increases epithelial permeability, thereby increasesing pro-inflammatory cytokine levels in the colon and LPS in the serum^39^. In addition, human studies demonstrated that subjects with high visceral adiposity and T2D have increased levels of bacterial DNA in their blood^40^.The present study showed that the composition of gut microbe-derived EVs significantly differs from that of gut microbes, and diet change from the RD to HFD regimen induced more drastic changes in the composition of bacterial EVs versus bacteria. In the present study, we also found that gut microbe-derived EVs, but not gut microbes, infiltrated through the intestinal barrier and entered the systemic circulation; then, they were distributed to insulin-responsive organs, such as liver, adipose tissue, and skeletal muscle. Moreover, this study demonstrated that gut microbe-derived EVs, enhanced by the HFD, induced insulin resistance in insulin-responsive organs, thereby impairing glucose metabolism. Considering the presence of LPS and DNA in bacterial EVs, these findings suggest that gut microbe-derived EVs are important messengers between gut microbes and host glucose metabolism.

The coevolution of gut microbiota and humans has conferred benefits to both the host and the bacterial community. However, dramatic changes in modern diets, especially high calorie HFDs, have resulted in the disruption of homeostasis between gut microbes and hosts, leading to insulin resistance and impaired glucose metabolism. While gut microbes are restricted to the gut, the secreted EVs can penetrate through the intestinal barriers and enter the systemic circulation; they are then distributed to insulin-responsive organs. The finding that gut microbe-derived EVs directly induce insulin resistance while impairing glucose metabolism in both skeletal muscle and adipose tissue provides an important clue to understanding the mechanism underlying impaired glucose metabolism in the pathogenesis of T2D.

## Additional Information

**How to cite this article**: Choi, Y. *et al.* Gut microbe-derived extracellular vesicles induce insulin resistance, thereby impairing glucose metabolism in skeletal muscle. *Sci. Rep.* **5**, 15878; doi: 10.1038/srep15878 (2015).

## Supplementary Material

Supplementary Information

Dataset 1
