# Supplementary material for: Gut microbe-derived extracellular vesicles induce insulin resistance, thereby impairing glucose metabolism in skeletal muscle
Source: Sci Rep. 2015 Oct 29;5:15878. doi: 10.1038/srep15878 (PMC4625370; doi:10.1038/srep15878)
Supplement: Supplementary Information [file srep15878-s1.pdf]

Supplementary Information

Gut microbe-derived extracellular vesicles induce insulin resistance, thereby impairing glucose metabolism in skeletal muscle

Youngwoo Choi,[1] Yonghoon Kwon,[1] Dae-Kyum Kim,[1] Jinseong Jeon,[1] Su Chul Jang,[1] Taejun Wang,[2] Byung Jun Kang, [1] Minjee Ban,[1] Min-Hye Kim,[3] Seong Gyu Jeon,[1] Min-Sun Kim,[4] Cheol Soo Choi,[5] Young-Koo Jee,[6] Yong Song Gho,[1] Sung-Ho Ryu,[1] and Yoon-Keun Kim[3]

[1]Department of Life Sciences, Pohang University of Science and Technology (POSTECH), Pohang; [2]Department of Interdisciplinary Biosciences and Biotechnology, POSTECH, Pohang; [3]Department of Medicine and Ewha Institute of Convergence Medicine, Ewha Womans University School of Medicine, Seoul; [4]Department of Internal Medicine, Ulsan University College of Medicine, Seoul; [5]Korea Mouse Metabolic Phenotyping Center, Lee Gil Ya Cancer and Diabetes Institute, and Division of Endocrinology, Gil Medical Center, Gachon University, Incheon; [6]Department of Internal Medicine, Dankook University College of Medicine, Cheonan, Republic of Korea

Correspondence: Yoon-Keun Kim (juinea@ewha.ac.kr)

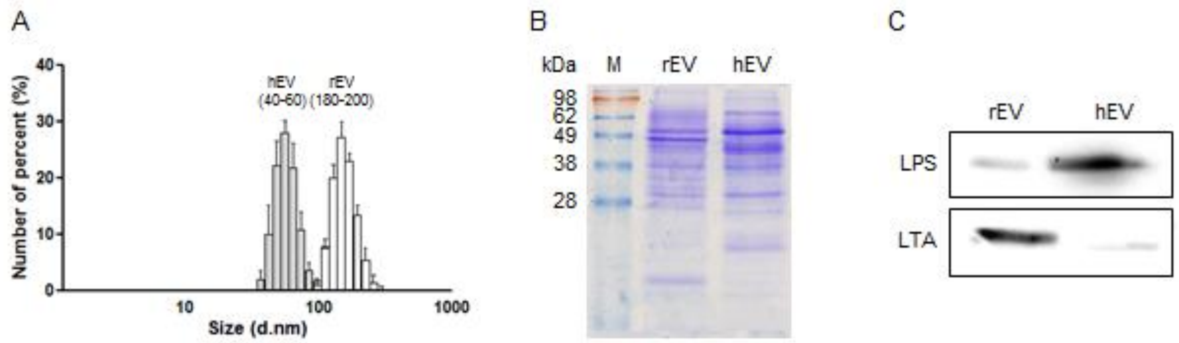

Figure S1. Characterization of stool EVs isolated from HFD- and RD-fed mice. (A) Size of hEV and rEV. Size was measured by using dynamic light scattering (DLS) method. (B) SDS-PAGE data of hEV and rEV. (C) Immunoblot analysis of lipid A and lipoteichoic acid of hEV and rEV.

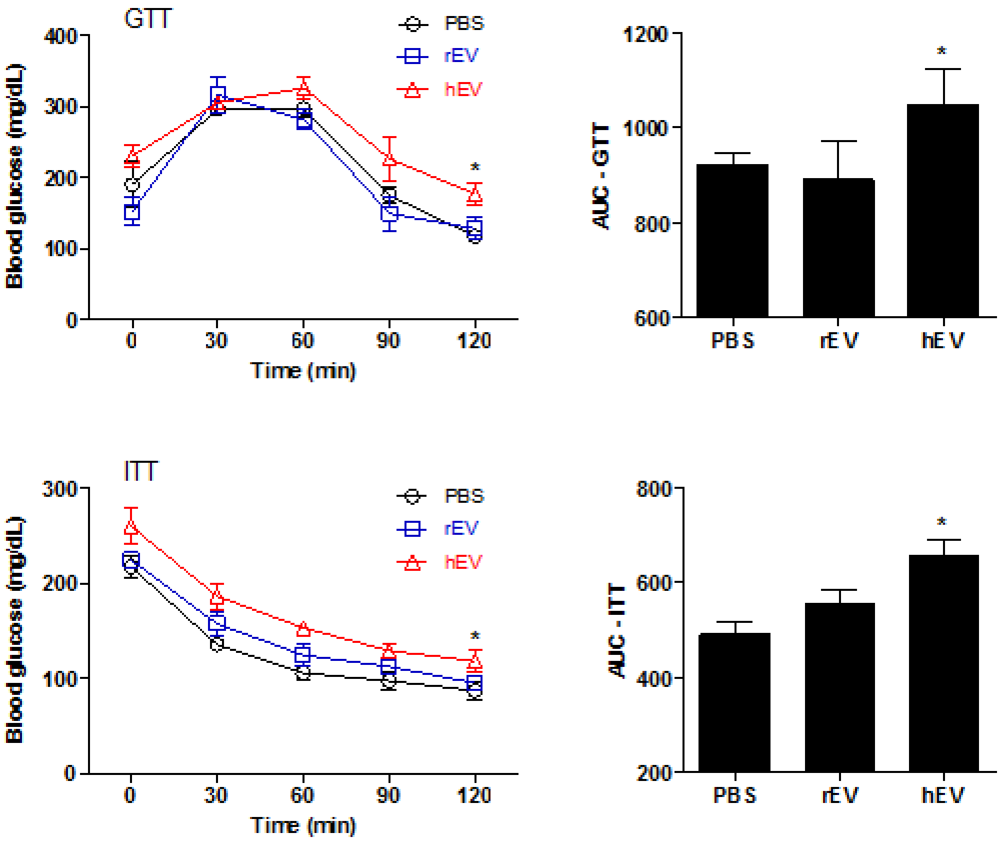

Figure S2. Glucose tolerance test (GTT) and insulin tolerance test (ITT) in RD-fed mice after the oral administration of hEV or rEV (n = 6 mice per group). *$P < 0.05$ vs. the other groups

Supplementary Table 1. The composition of common bacteria and bacteria-derived EVs in stools at the phylum, class, order, and family levels

Phylum		Bacteria (%)		EV (%)	
		RD	HFD	RD	HFD
Gram +	Firmicutes	31.6328	41.7077	3.02559	2.61536
	TM7	23.20219	11.51675	27.72303	7.17282
	Actinobacteria	3.76135	4.56347	1.64246	4.30887
	Tenericutes	5.79334	2.55794	0.51003	0.09322
Gram -	Proteobacteria	10.66436	25.83163	65.84544	84.03335
	Bacteroidetes	24.49921	13.72643	1.24481	1.67797

Class		Bacteria (%)		EV (%)	
		RD	HFD	RD	HFD
Gram +	TM7_c	23.18778	11.51675	27.72303	7.17282
	Clostridia	28.77936	6.07662	2.93914	0.90631
	Bacilli	1.28261	11.52876	0.08645	1.61065
	Mollicutes	5.79334	2.55794	0.51003	0.09322
	Actinobacteria_c	2.52198	0.75657	1.62517	4.2778
Gram -	Gammaproteobacteria	9.06471	23.47784	64.21162	79.18069
	Bacteroidia	24.42715	13.49826	0.90768	0.66808
	Alphaproteobacteria	0.27381	0.50438	0.98548	3.06593
	Betaproteobacteria	0.80703	0.42032	0.64834	1.78155

Order		Bacteria (%)		EV (%)	
		RD	HFD	RD	HFD
Gram +	EU381732_o	22.97161	11.09643	27.56743	7.10032
	Clostridiales	28.30379	5.94452	2.83541	0.90113
	Lactobacillales	0.87909	11.43269	0.0778	1.42939
	AM275436_o	5.69246	2.54593	0.50138	0.09322
	TM7_c_uc	0.18735	0.42032	0.14696	0.06733
Gram -	Pseudomonadales	8.22885	21.85661	62.0332	75.23435
	Bacteroidales	24.26863	13.43821	0.89903	0.65255
	Caulobacterales	0.21617	0.38429	0.67427	2.47035
	Xanthomonadales	0.69174	0.94872	1.17566	1.69869
	Burkholderiales	0.77821	0.3963	0.64834	1.70905
	Enterobacteriales	0.10088	0.60046	0.82988	2.02496

Family		Bacteria (%)		EV (%)	
		RD	HFD	RD	HFD
Gram +	EU381732_f	22.29428	10.6521	27.25622	6.9087
	Ruminococcaceae	20.65139	1.60922	2.02282	0.27448
	Lachnospiraceae	1.80141	2.13762	0.16425	0.23305
	FJ367060_f	5.21689	2.30575	0.1556	0.07251
	Clostridiales_uc	3.27136	0.87667	0.51867	0.14501
	EU381732_o_uc	0.67733	0.44434	0.3112	0.19162
	TM7_c_uc_f	0.18735	0.42032	0.14696	0.06733
Gram -	Pseudomonadaceae	8.07033	21.67647	61.72199	75.0738
	Caulobacteraceae	0.21617	0.36027	0.67427	2.44446
	Xanthomonadaceae	0.67733	0.84064	1.15837	1.69869
	Enterobacteriaceae	0.08647	0.56443	0.82123	2.02496
	Bacteroidales_uc	0.90791	0.91269	0.05187	0.05697
	Oxalobacteraceae	0.15852	0.2642	0.35443	0.94257
	Pseudomonadales_uc	0.15852	0.16813	0.3112	0.13983

Supplementary Table 2. Genus level composition of common bacteria and bacteria-derived EVs in stools

Genus		Bacteria (%)		EV (%)	
		RD	HFD	RD	HFD
Gram +	EU381732_g	20.36316	8.91077	24.55048	5.83148
	4P000387_g	4.88543	2.08959	0.11238	0.04143
	EU381732_f_uc	1.55642	1.38105	2.03147	0.96328
	Clostridiales_uc_g	3.27136	0.87667	0.51867	0.14501
	Ruminococcaceae_uc	2.70932	0.24018	0.27663	0.10358
	EU381732_o_uc_g	0.67733	0.44434	0.3112	0.19162
	Lachnospiraceae_uc	0.69174	0.76858	0.04322	0.06215
	EF445280_g	0.37469	0.36027	0.67427	0.11394
	TM7_c_uc_g	0.18735	0.42032	0.14696	0.06733
	EU509847_g	0.21617	0.14411	0.26798	0.01036
	FJ367060_f_uc	0.33146	0.21616	0.04322	0.03107
	Rhodococcus	0.01441	0.02402	0.01729	0.08804
Gram -	Pseudomonas	7.75328	21.38826	61.5491	74.91325
	Brevundimonas	0.17294	0.33626	0.59647	2.36159
	Escherichia	0.05765	0.49237	0.7175	1.74012
	Alistipes	1.32584	0.72055	0.05187	0.02072
	Stenotrophomonas	0.04323	0.20416	0.31985	1.47082
	Xanthomonadaceae_uc	0.56204	0.55242	0.68292	0.1709
	Bacteroidales_uc_g	0.90791	0.91269	0.05187	0.05697
	Elizabethkingia	0.04323	0.15612	0.30256	0.95292
	Janthinobacterium	0.11529	0.15612	0.31985	0.83899
	Pseudomonadaceae_uc	0.31705	0.28822	0.17289	0.16055
	Pseudomonadales_uc_g	0.15852	0.16813	0.3112	0.13983
	Delftia	0.02882	0.03603	0.09509	0.35217
	FJ184353_g	0.07206	0.08406	0.1556	0.05697
	Gammaproteobacteria_uc_g	0.04323	0.06005	0.1556	0.05179
	Caulobacteraceae_uc	0.04323	0.02402	0.0778	0.06215
	Oxalobacteraceae_uc	0.02882	0.08406	0.02593	0.06215
	Enterobacteriaceae_uc	0.02882	0.04804	0.02593	0.04143

Supplementary Table 3. Species level composition of common bacteria and bacteria-derived EVs in stools

Genus		Bacteria (%)		EV (%)	
		RD	HFD	RD	HFD
	AJ400239_s	13.60427	0.76858	14.54876	0.78202
	DQ777900_s	4.89984	3.20644	7.40837	2.69822
	EU381732_g_uc	1.81582	4.93575	2.55014	2.3357
	4P000387_s	4.23692	2.07758	0.11238	0.03107
	EU381732_f_uc_s	1.55642	1.38105	2.03147	0.96328
	Clostridiales_uc_s	3.27136	0.87667	0.51867	0.14501
	Ruminococcaceae_uc_s	2.70932	0.24018	0.27663	0.10358
Gram +	EU381732_o_uc_s	0.67733	0.44434	0.3112	0.19162
	Lachnospiraceae_uc_s	0.69174	0.76858	0.04322	0.06215
	EU469875_s	0.30264	0.27621	0.59647	0.09322
	TM7_c_uc_s	0.18735	0.42032	0.14696	0.06733
	FJ367060_f_uc_s	0.33146	0.21616	0.04322	0.03107
	EU509847_s	0.02882	0.07205	0.24205	0.01036
	EF445280_g_uc	0.07206	0.08406	0.0778	0.02072
	Rhodococcus qingshengii group	0.01441	0.02402	0.01729	0.08286
	Pseudomonas extremaustralis	3.79017	12.42945	15.30083	28.57735
	Pseudomonas cedrina	0.1297	0.01201	27.6971	5.93506
	Pseudomonas panacis	1.72936	5.42813	6.94156	14.16438
	Pseudomonas trivialis	1.13849	2.00552	5.73997	17.97607
	Pseudomonas grimontii	0.2594	0.07205	1.69433	3.34559
	Pseudomonas libanensis	0.10088	0.06005	2.32538	1.11865
	Escherichia coli group	0.04323	0.45635	0.68292	1.66762
	Brevundimonas vesicularis group	0.11529	0.21616	0.389	1.66244
	Xanthomonadaceae_uc_s	0.56204	0.55242	0.68292	0.1709
	Bacteroidales_uc_s	0.90791	0.91269	0.05187	0.05697
	Pseudomonas proteolytica	0.07206	0.46836	0.40629	0.8597
	Elizabethkingia miricola	0.04323	0.15612	0.29391	0.94774
	Pseudomonas hibiscicola	0.04323	0.10808	0.24205	1.03579
	Pseudomonas marginalis	0.07206	0.03603	0.28527	0.91667
	Pseudomonas brenneri	0.11529	0.34826	0.27663	0.42985
Gram -	Pseudomonas_uc	0.11529	0.18014	0.34578	0.45057
	Brevundimonas intermedia	0.04323	0.12009	0.16425	0.64219
	Pseudomonadaceae_uc_s	0.31705	0.28822	0.17289	0.16055
	Pseudomonadales_uc_s	0.15852	0.16813	0.3112	0.13983
	Janthinobacterium lividum	0.05765	0.06005	0.18154	0.43503
	AF430121_s	0.01441	0.02402	0.1556	0.42985
	Janthinobacterium agaricidamnosum	0.02882	0.09607	0.11238	0.36253
	Delftia lacustris	0.02882	0.03603	0.0778	0.2952
	FJ184353_g_uc	0.07206	0.08406	0.1556	0.05179
	Gammaproteobacteria_uc_s	0.04323	0.06005	0.1556	0.05179
	Pseudomonas palleroniana	0.11529	0.02402	0.06916	0.01554
	Pseudomonas baetica	0.01441	0.06005	0.06916	0.06733
	Caulobacteraceae_uc_s	0.04323	0.02402	0.0778	0.06215
	Oxalobacteraceae_uc_s	0.02882	0.08406	0.02593	0.06215
	Enterobacteriaceae_uc_s	0.02882	0.04804	0.02593	0.04143
	Pseudomonas migulae	0.01441	0.02402	0.03458	0.04661
	Escherichia_uc	0.01441	0.01201	0.03458	0.05697

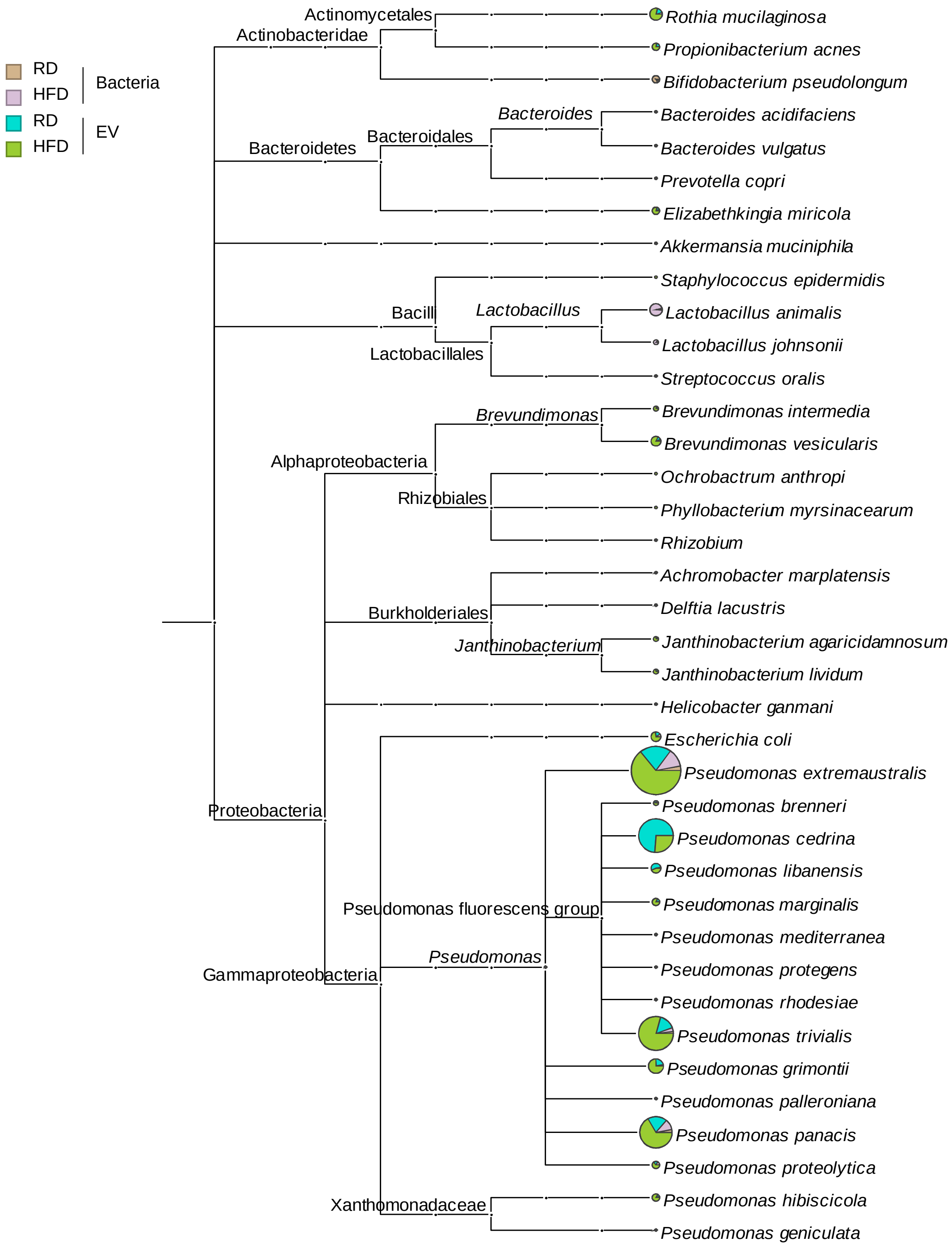

Figure S3. 16S rDNA gene sequencing shows proportional changes in bacteria and bacteria-derived EVs in mice fed with a HFD for 12 weeks. This figure shows the hierarchical clustering plot of bacteria and bacteria-derived EVs in stools.

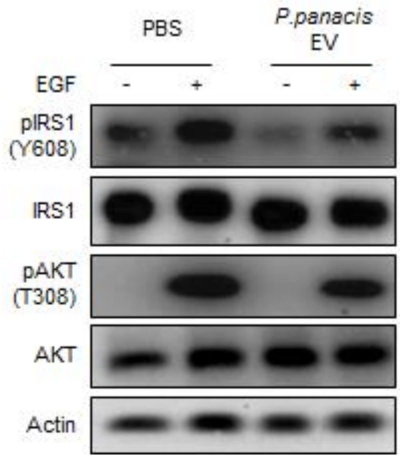

Figure S4. *P. panacis* EVs do not interfere with the EGF-signaling pathway. Western blot data of EGF-signaling molecules in L6 myotubes treated with *P. panacis* EVs.

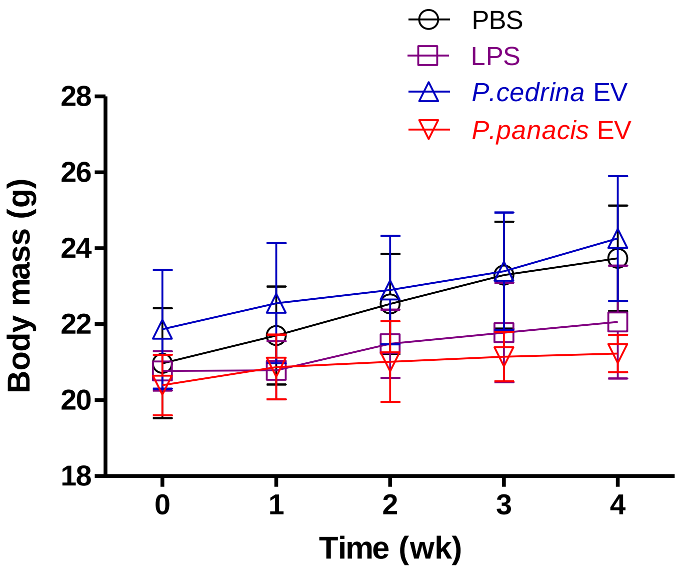

Figure S5. Body weight change after the oral administration of LPS, *P. cedrina* EVs, or *P. panacis* EVs (n = 5 mice per group).
